# High oral carriage of multidrug resistant Gram-negative bacilli in adolescents: the SOPKARD-Junior study

**DOI:** 10.3389/fcimb.2023.1265777

**Published:** 2023-11-16

**Authors:** Marta Katkowska, Katarzyna Garbacz, Ewa Kwapisz, Klaudia Suligowska, Aida Kusiak, Dominika Cichońska, Dariusz Świetlik

**Affiliations:** ^1^ Department of Oral Microbiology, Faculty of Medicine, Medical University of Gdansk, Gdansk, Poland; ^2^ Department of Dental Techniques and Masticatory System Dysfunctions, Faculty of Medicine, Medical University of Gdansk, Gdansk, Poland; ^3^ Department of Preventive Medicine and Education, Faculty of Medicine, Medical University of Gdansk, Gdansk, Poland; ^4^ Department of Periodontology and Oral Mucosa Diseases, Faculty of Medicine, Medical University of Gdansk, Gdansk, Poland; ^5^ Division of Biostatistics and Neural Networks, Medical University of Gdansk, Faculty of Medicine, Medical University of Gdansk, Gdansk, Poland

**Keywords:** multidrug resistant (MDR), Gram-negative bacilli (GNB), *Enterobacter*, *Pseudomonas*, *Serratia*, extended-spectrum β-lactamase (ESBL), oral carriage, oral colonization

## Abstract

**Introduction:**

The colonization of the oral cavity by potentially pathogenic antimicrobial-resistant bacteria in adolescents and its consequences is very poorly understood. The present study focused on the occurrence of oral colonization by Gram-negative bacilli (GNB) and their multidrug resistance, including the production of extended-spectrum β-lactamases (ESBLs) and carbapenemases, among healthy adolescents and risk factors associated with GNB colonization.

**Materials and methods:**

This study was conducted as part of “A program for the early detection of risk factors for lifestyle diseases SOPKARD-Junior” (SOPKARD-Junior). Oral samples were collected from 182 adolescents from four public elementary schools in Sopot, Poland, aged 13-14 years. Bacterial strains were identified by the MALDI-TOF MS method. Screening of antimicrobial resistance was performed using a disk diffusion method. The NG-Test^®^ CARBA-5 was used to detect and differentiate the five most widely distributed carbapenemases. Demographic and clinical data were collected and statistical analysis of risk factors was performed.

**Results:**

A total of 68 out of 182 (37.4%) healthy adolescents was documented oral colonization with Gram-negative bacilli, including 50/182 (27.5%) multidrug resistant (MDR-GNB) strains. Over 60% of oral carriage concerned three main genera *Enterobacter* spp., *Pseudomonas* spp., and *Serratia* spp., which were detected in 22.1%, 19.1%, and 19.1% of participants, respectively. *Citrobacter* spp., *Escherichia coli*, *Klebsiella* spp., *Hafnia* spp., *Aeromonas* spp., *Acinetobacter* spp., and *Stenotrophomonas* spp. were also isolated. The antimicrobial resistance to ampicillin (100%), ceftazidime (69.1%), meropenem (60.3%), gentamycin (60.3%), piperacillin/tazobactam (52.9%), and piperacillin (45.6%) were the most common. Among 73.5% GNB strains multidrug resistance was observed, including all *Pseudomonas* spp. strains. Among MDR-GNB, 30.4% were resistant to four groups of antibiotics, half of the MDR *Pseudomonas* spp. strains were resistant to 10 groups of antibiotics. Extended-spectrum β-lactamases were produced by *Enterobacter cloacae*, *Klebsiella* spp., and *Serratia* spp. (7.4%). Colonization by ESBLs-positive GNB strains was significantly associated with recurrent respiratory infections, nasal congestion, and bronchitis (p<0.05).

**Conclusion:**

Our study revealed high oral carriage of multi-drug resistant Gram-negative bacilli in healthy adolescents and the association of ESBL-producing strains with respiratory infections. Further studies on oral colonization with GNB are necessary due to the possibility of distinct infections and the acquisition of antibiotic resistance by resident microbiota.

## Introduction

The oral cavity is one of the most biologically complex niches in the body and the body’s entrance to the outside world. In particular from the air or through ingestion while eating, the mouth can serve as a site of entry for various microbial pathogens. Specific features of the oral cavity, such as specialized mucosal surfaces, teeth enamel, and salivary flow, make it distinct from all other body surfaces leading to colonization by selected microbes. As a consequence, not all microorganisms that enter the mouth can persist and form the oral microbiota. The composition of the oral microflora is dynamic and changes over human life. The microbial community of the mouth coexists harmoniously with the host, and this symbiosis is advantageous to both. Exogenous and commonly pathogenic bacteria can colonize the mouth as a result of the loss or disruption of resident microbiota, predisposing to many disorders (Jakubovics, 2015; Marsh et al., 2016).

Oral carriage of both Gram-positive and -negative bacteria such as *Streptococcus pneumoniae*, *Streptococcus pyogenes*, *Staphylococcus aureus*, *Haemophilus influenzae*, *Moraxella catarrhalis*, especially in children, is very well known, but there are only a few studies reported the oral colonization by Gram-negative bacilli (GNB) (Lima et al., 2010; Le et al., 2020; Osei et al., 2022; Leão et al., 2023). The persistence of GNB in the oropharyngeal area makes patients, primarily hospitalized or immunocompromised, vulnerable to bacterial infections such as pneumonia, bacteremia, and urinary tract infections (Osei et al., 2022). The hazard of GNB colonization is mainly due to their widespread resistance to antibiotics including the production of extended-spectrum β-lactamases (ESBLs) and carbapenemases. Gram-negative bacilli such as *Acinetobacter baumannii*, *Pseudomonas aeruginosa*, and *Enterobacter* spp. is one of the most frequent causes of nosocomial infections belonging to the alarming ESKAPE group (Paauw et al., 2009; Akbari et al., 2016; Motiwala et al., 2022).

While previous studies evaluated Gram-negative bacterial carriage mainly based on fecal or rectal samples, recent studies indicated the presence of GNB in the oropharyngeal region in addition to the other sites revealed striking differences in GNB colonization rates depending on sampling protocols, selected body sites for screening, and microbiological testing procedures (Le et al., 2020). Just as there is a consensus on sampling methods for the carriage of some bacteria (e.g. nasal swab collection for methicillin-resistant *S. aureus*), there is debate over the collection of screening samples for some MDR Gram-negative bacteria (Torres et al., 2022).

For these reasons, the study conducted within the SOPKARD-Junior program addresses the prevalence of oral colonization by multidrug-resistant GNB, including extended-spectrum β-lactamases (ESBLs)- and carbapenemases-producing strains, among healthy adolescents and risk factors associated with GNB colonization.

## Materials and methods

### Study design and participants

The study was carried out as part of a preventive program “A program for the early detection of risk factors for lifestyle diseases SOPKARD-Junior” (SOPKARD-Junior). The main purpose of the program was to assess the health condition of adolescents. The program was conducted from September to December 2021 in all elementary schools in the city of Sopot in the Pomeranian Voivodeship of Poland. All teenagers (n=246) aged 13-14 years attending 8th grade of Sopot elementary schools (attending school regularly, without long breaks due to hospitalization or chronic diseases) were invited to the SOPKARD-Junior program. The study began after receiving written informed consent from each child’s parent or legal guardian, as well as approval from the bioethics committee of the Medical University of Gdansk (NKBBN/510-745/2021). A total of 182 participants took part in the microbiological study. Adolescents did not eat or drink or brush their teeth for a minimum of one hour before sample collection. One oral sample was collected from each study participant using a sterile swab on STUART transport medium (MEDLAB-PRODUCTS, Raszyn, Poland) and directly transported to the laboratory of the Department of Oral Microbiology of the Medical University of Gdansk.

The data such as sex, prevalence of comorbidities such as bronchitis, otitis, tonsillitis, tonsillectomy, adenoid hypertrophy, chronic rhinitis, allergic rhinitis, nasal congestion, sinusitis, asthma, recurrent respiratory infections, allergy, atopic dermatitis, psoriasis, thyroid disease, liver disease, kidney disease, cardiologic disease, diabetes, hypercholesterolemia, attention deficit hyperactivity disorder (ADHD), and depression were collected.

### Bacterial strains and screening of antimicrobial resistance

A total of 182 oral swabs were collected. All swabs were subcultured on Columbia blood agar (GrasoBiotech, Starogard Gd., Poland) and MacConkey agar (bioMérieux, Marcy l’Etoile, France) and incubated aerobically at 35 ± 2°C for 18 to 24h. Bacterial strains were identified according to standard procedure routine microbiological diagnostics, including the API system (bioMeriux, Marcy-l’Etoile, France) (Tille, 2013). Identification of GNB species was verified by the matrix-assisted laser desorption ionization time-of-flight mass spectrometry (MALDI-TOF MS) method (Bruker Daltonics, MA, USA).

The screening of antimicrobial susceptibility of isolated bacteria was performed by disk diffusion method according to the European Committee on Antimicrobial Susceptibility Testing (EUCAST) (EUCAST, 2021). In total, the used antimicrobial agents included amikacin (30 µg), ampicillin (10 µg), aztreonam (30 µg), cefoxitin (30 µg), cefepime (30 µg), ceftazidime (10 µg), ciprofloxacin (5 µg), colistin (10 µg), gentamycin (10 µg), imipenem (10 µg), levofloxacin (5 µg), meropenem (10 µg), piperacillin (30 µg), piperacillin/tazobactam (30µg/6µg), ticarcillin (75 µg), ticarcillin/clavulanic acid (75µg/10µg), trimethoprim/sulfamethoxazole (1.25µg/23.75µg) (Oxoid, Basingstoke, England). The multidrug resistant Gram-negative bacilli (MDR-GNB) were defined as strains resistant to one or more agents in three or more antimicrobial categories.

The strains produced of extended-spectrum β-lactamases (ESBL) were detected with discs with ceftazidime (30 µg), cefotaxime, and aztreonam (30 µg) placed at a distance of 20 mm from the center of the disc with amoxicillin/clavulanic acid (20 µg/10 µg) (EUCAST, 2022).

CHROMagar ESBL (GrasoBiotech, POLAND) and NG-Test^®^ CTX-M MULTI (NG BIOTECH, France) were used to confirm the occurrence of the ESBL mechanism in the tested positive strains. The test allows detection of the five major groups (1, 2, 8, 9 and 25) in the CTX-M-type enzymes of extended-spectrum β-lactamases (ESBLs). The NG-Test^®^ CARBA-5 (NG BIOTECH, France) was used to detect carbapenemase-producing strains. The test determined the five most widely distributed carbapenemases (KPC, NDM, IMP, VIM, OXA-48) belonging to the following classes: A - *Klebsiella pneumoniae* carbapenemase (KPC); class B - New Delhi metallo-β-lactamase (NDM), imipenemase (IMP), Verona integron–encoded metallo-β-lactamase (VIM), and class D - oxacillinase (OXA-48).

### Statistical analysis

The analysis of association between the demographic/clinical data of participants and occurrence of antimicrobial-resistant GNB was performed. Statistical analysis was performed using software from TIBCO Software Inc. (2017) Statistica (data analysis software system), version 13. http://statistica.io. The qualitative variables were presented with the use of count and percentage. Chi-squared tests for independence were used for qualitative variables. In all calculations, α=0.05 was taken as the significance level.

## Results

### Distribution of Gram-negative bacilli

**Figure 1:**
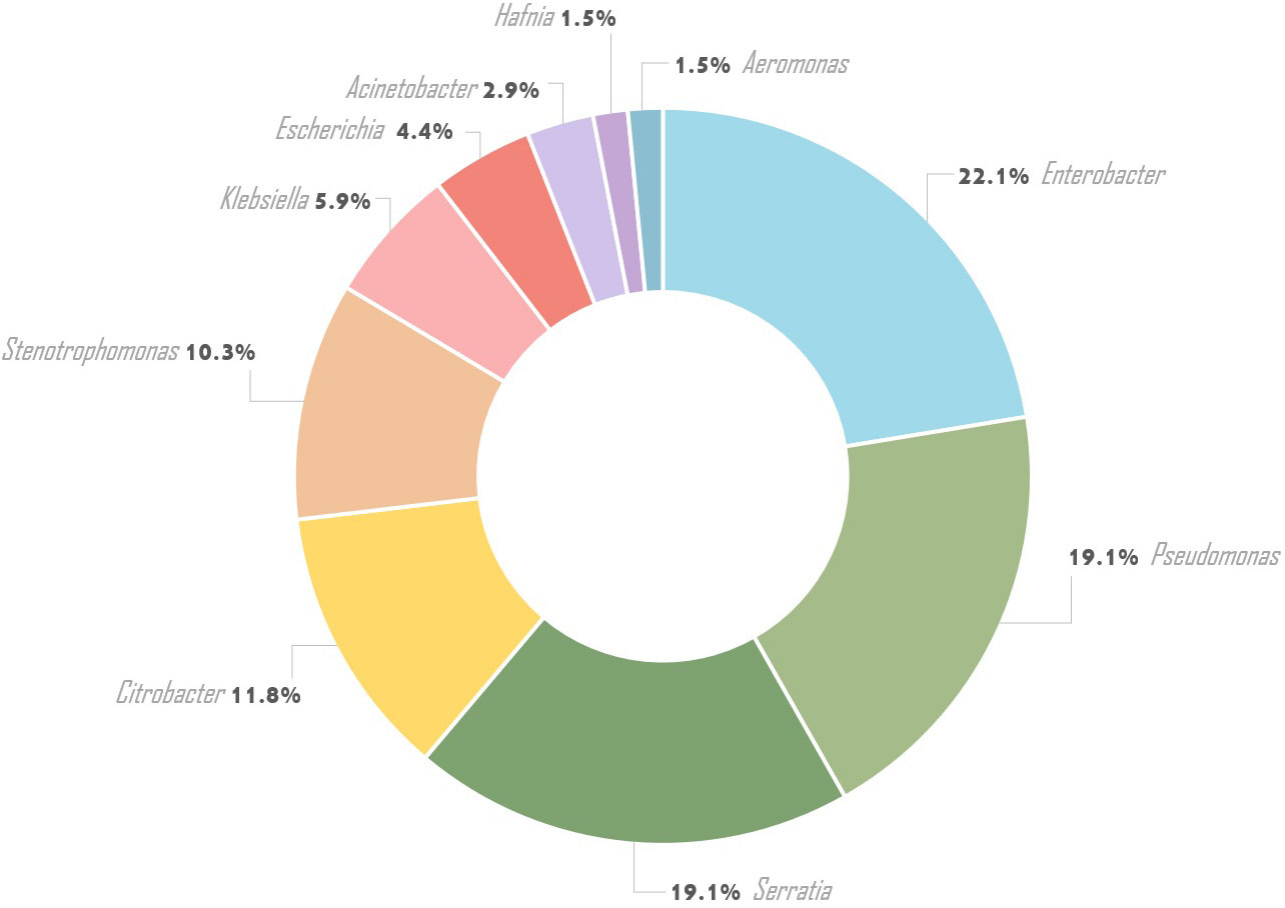
Prevalence of GNB isolation from the oral cavity.

From 182 oral swabs collected from healthy adolescents, 68 (one strain from each person) Gram-negative bacilli were isolated (37.4%), including Enterobacterales (46/68 - 67.6%) and non-fermenting bacilli strains (22/68 - 32.4%). Ten bacterial species belonging to the Enterobacterales comprised *Enterobacter cloacae* (15/46 – 32.6%), *Serratia marcescens* (11/46 – 23.9%), *Citrobacter freundii* (7/46 – 15.2%), *Escherichia coli* (3/46 – 6.5%), *Klebsiella oxytoca* (3/46 – 6.5%), *Serratia odorifera* (2/46 – 4.3%), *Citrobacter koseri* (2/46 – 4.3%), *Hafnia alvei* (1/46 – 2.2%), *Aeromonas hydrophila* (1/46 – 2.2%), and *Klebsiella pneumoniae* (1/46 – 2.2%). Ten non-fermenting bacilli species such as *Pseudomonas aeruginosa* (7/22 – 31.8%), *Stenotrophomonas maltophilia* (7/22 – 31.8%), *Pseudomonas tolaasii* (1/22 – 4.5%), *Pseudomonas extremorientalis* (1/22 – 4.5%), *Pseudomonas jessenii* (1/22 – 4.5%), *Pseudomonas rhodesiae* (1/22 – 4.6%), *Pseudomonas monteilii* (1/22 – 4.6%), *Pseudomonas lundensis* (1/22 – 4.5%), *Acinetobacter radioresistens* (1/22 – 4.5%), and *Acinetobacter baumanii* (1/22 – 4.5%) were identified (**Table 1**; **Figure 1**).

**Table 1:** The frequency of Gram-negative bacilli species isolated from the oral cavity of healthy adolescents.

Bacterial species	Enterobacterales (n=46) N (%)	Gram-negative bacilli (n=68) N (%)
***Enterobacter cloacae***	15 (32.6)	15 (22.1)
***Serratia marcescens***	11 (23.9)	11 (16.2)
***Citrobacter freundii***	7 (15.2)	7 (10.3)
***Escherichia coli***	3 (6.5)	3 (4.4)
***Klebsiella oxytoca***	3 (6.5)	3 (4.4)
**Serratia odorifera**	2 (4.3)	2 (2.9)
***Citrobacter koseri***	2 (4.3)	2 (2.9)
***Hafnia alvei***	1 (2.2)	1 (1.5)
***Aeromonas hydrophila***	1 (2.2)	1 (1.5)
***Klebsiella pneumoniae***	1 (2.2)	1 (1.5)
Bacterial species	Non-fermenting bacilli (n=22) n (%)	Gram-negative bacilli (n=68) n (%)
***Pseudomonas aeruginosa***	7 (31.8)	7 (10.3)
***Stenotrophomonas maltophilia***	7 (31.8)	7 (10.3)
***Pseudomonas tolaasii***	1 (4.5)	1 (1.5)
***Pseudomonas extremorientalis***	1 (4.5)	1 (1.5)
***Pseudomonas jessenii***	1 (4.5)	1 (1.5)
***Pseudomonas rhodesiae***	1 (4.5)	1 (1.5)
***Pseudomonas monteilii***	1 (4.5)	1 (1.5)
***Pseudomonas lundensis***	1 (4.5)	1 (1.5)
***Acinetobacter radioresistens***	1 (4.5)	1 (1.5)
***Acinetobacter baumanii***	1 (4.5)	1 (1.5)

### Antimicrobial resistance

**Figure 2:**
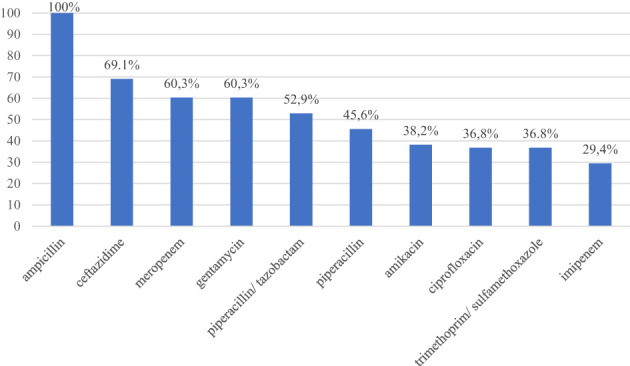
Antibiotic resistance of Gram-negative bacilli isolated from the oral cavity of healthy adolescents.

According to the EUCAST recommendation, all isolated GNB strains were resistant to ampicillin (100%), followed by resistance to ceftazidime (69.1%), meropenem (60.3%), gentamycin (60.3%), piperacillin/tazobactam (52.9%), piperacillin (45.6%), amikacin (38.2%), ciprofloxacin (36.8%), trimethoprim/sulfamethoxazole (36.8%), and imipenem (29.4%) (**Table 2**; **Figure 2**). All *Pseudomonas* spp. strains were resistant to amikacin, ciprofloxacin, imipenem, aztreonam, piperacillin, piperacillin/tazobactam, ticarcillin and levofloxacin. Resistance of *Pseudomonas* spp. strains to cefepime, ceftazidime and ticarcillin/clavulanic acid was 92.3%, to colistin 84.6% and to meropenem 53.9%.

**Table 2:** Antibiotic resistance of Gram-negative bacilli isolated from the oral cavity (by genus).

	*Enterobacter* spp. (n=15) N (%)	*Serratia* spp. (n=13) N (%)	*Citrobacter* spp. (n=9) N (%)	*Klebsiella* spp. (n=4) N (%)	*E. coli* spp. (n=3) N (%)	*Aeromonas* spp. (n=1) N (%)	*Hafnia* spp. (n=1) N (%)	*Pseudomonas* spp. (n=13) N (%)
**AKN**	0	2 (15.4)	0	1 (25)	2 (66.7)	1 (100)	0	13 (100)
**AMP**	15 (100)	13 (100)	9 (100)	4 (100)	3 (100)	1 (100)	1 (100)	NT
**CIP**	2 (13.3)	1 (7.7)	0	0	0	0	0	13 (100)
**CZD**	12 (80)	2 (15.4)	7 (77.8)	2 (50)	1 (33.3)	1 (100)	1 (100)	12 (92.3)
**GMN**	6 (40)	0	8 (88.9)	2 (50)	3 (100)	1 (100)	1 (100)	NT
**IMP**	0	0	0	0	0	0	0	13 (100)
**MEM**	11 (73.3)	9 (69.2)	1 (11.1)	1 (25)	3 (100)	1 (100)	1 (100)	7 (53.9)
**PIL**	2 (13.3)	2 (15.4)	0	3 (75)	1 (33.3)	1 (100)	0	13 (100)
**PTZ**	7 (46.7)	2 (15.4)	1 (11.1)	2 (50)	1 (33.3)	1 (100)	0	13 (100)
**SXT**	0	2 (15.4)	1 (11.1)	0	0	0	0	NT

AKN-amikacin; AMP-ampicillin; CIP-ciprofloxacin; CZD- ceftazidime; GMN-gentamycin; IMP-imipenem; MEM-meropenem; PIL- piperacillin; PTZ- piperacillin/tazobactam; SXT- trimethoprim/sulfamethoxazole; NT- not tested.

Out of 68 GNB strains, 5 (7.4%) produced extended-spectrum β-lactamases, *Enterobacter* spp. (3/68), *Klebsiella* spp. (1/68), and *Serratia* spp. strains (1/68). None of the isolated GNB strains produced carbapenemases. Fifty (73.5%) multidrug-resistant GNB were identified, including all *Pseudomonas* spp. and *Stenotrophomonas maltophilia* strains (29.4%), and Enterobacterales strains (44.1%). Among MDR-GNB, 30.4% were resistant to four classes of antimicrobials, half of the MDR *Pseudomonas* spp. strains were resistant to 10 classes of antimicrobials.

### Demographic and clinical characteristic

The analysis of demographic and clinical data showed that most of them (sex, school, bronchitis, otitis, tonsillitis, tonsillectomy, adenoid hypertrophy, chronic rhinitis, nasal congestion, sinusitis, asthma, recurrent respiratory infections, allergy, psoriasis, thyroid disease, liver disease, kidney disease, cardiologic disease, diabetes, hypercholesterolemia, ADHD and depression) did not affect growth of oral colonization by GNB. Only atopic dermatitis (p=0.008) and allergic rhinitis (p=0.029) revealed statistically significant differences between colonized and non-colonized adolescents (**Table 3**). The prevalence of ESBL-positive strains was significantly associated with participants reporting recurrent respiratory infections (p=0.004), nasal congestion (p=0.008), and bronchitis (p=0.045). Whereas, no association was found between the analyzed data and MDR *Pseudomonas* spp. colonization (**Table 4**).

**Table 3:** Characteristics of the studiedadolescents with and without oral carriage of Gram-negative bacilli.

Characteristics	No colonization (N = 114) No (%)	Colonization (N = 68) No (%)	p-value
School			
SP1 (n=14)	10 (71.4)	4 (28.6)	0.4791
SP7 (n=55)	33 (64.7)	18 (35.3)	0.7189
SP8 (n=51)	35 (56.5)	27 (43.5)	0.2150
SP9 (n=62)	36 (65.5)	19 (34.5)	0.6052
Sex			
Male (105)	70 (61.4)	35 (51.5)	0.1895
Female (77)	44 (38.6)	33 (48.5)	

Bronchitis	23 (20.2)	14 (20.6)	0.9466
Otitis	18 (15.8)	7 (10.3)	0.3183
Tonsillitis	16 (14.0)	6 (8.8)	0.2968
Tonsillectomy	7 (6.1)	4 (5.9)	0.9437
Adenoid hypertrophy	6 (5.3)	4 (5.9)	0.8592
Chronic rhinitis	16 (14.0)	13 (19.1)	0.3648
Nasal congestion	16 (14.0)	13 (19.1)	0.3648
Allergic rhinitis	4 (3.5)	8 (11.8)	0.0299
Sinusitis	10 (8.8)	5 (7.4)	0.7363
Asthma	7 (6.1)	1 (1.5)	0.1371
Recurrent respiratory infections	6 (5.3)	2 (2.9)	0.4598
Allergy	4 (3.5)	5 (7.4)	0.2472
Atopic dermatitis	0	4 (5.9)	0.0088
Psoriasis	0	1 (1.5)	0.1942
Thyroid disease	4 (3.5)	1 (1.5)	0.4158
Liver disease	2 (1.8)	1 (1.5)	0.8843
Kidney disease	1 (0.9)	1 (1.5)	0.7103
Cardiologic disease	1 (0.9)	0	0.4387
Diabetes	1 (0.9)	0	0.4387
Hypercholesterolaemia	0	1 (1.5)	0.1942
ADHD	1 (0.9)	1 (1.5)	0.7103
Depression	0	1 (1.5)	0.1942

ADHD - attention deficit hyperactivity disorder.

**Table 4:** Characteristics of the studies adolescents with oral carriage of ESBL-producing Enterobacterales and MDR *Pseudomonas* strains.

Characteristics	Enterobacterales (n=46)			Non-fermenting bacilli (n=22)		
	ESBL-negative (n=41)	ESBL-positve (n=5)	p value	MDR *Pseudomonas* (n=13)	Other (n=9)	p value
School						
SP7	7 (15.2)	1 (2.2)	0.8326	6 (27.3)	5 (22.7)	0.4664
SP9	18 (39.1)	2 (4.3)	0.9698	4 (18.2)	3 (13.6)	0.7507
SP8	15 (32.6)	2 (4.3)	0.8209	2 (9.1)	0	0.2435
SP1	3 (6.5)	0	0.5419	1 (4.5)	0	0.4215
Sex						
Male	22 (47.8)	3 (6.5)	0.7469	6 (27.3)	4 (18.2)	0.8639
Female	20 (43.5)	2 (4.3)		7 (31.8)	4 (18.2)		

Bronchitis	8 (17.4)	3 (6.5)	0.0451	3 (13.6)	0		0.1210
Otitis	4 (8.7)	0	0.4648	2 (9.1)	1 (4.5)		0.7740
Tonsillitis	5 (10.9)	0	0.4082	1 (4.5)	0		0.3944
Tonsillectomy	2 (4.3)	1 (2.2)	0.1961	0	1 (4.5)		0.2367
Adenoid hypertrophy	2 (4.3)	0	0.6136	2 (9.1)	0		0.2172
Chronic rhinitis	6 (13.0)	1 (2.2)	0.7525	4 (18.2)	2 (9.1)		0.6581
Nasal congestion	5 (10.9)	3 (6.5)	0.0078	4 (18.2)	1 (4.5)		0.2367
Allergic rhinitis	4 (8.7)	1 (2.2)	0.4872	2 (9.1)	1 (4.5)		0.7740
Sinusitis	2 (4.3)	1 (2.2)	0.1961	1 (4.5)	1 (4.5)		0.7839
Asthma	1 (2.2)	0	0.7240	0	0		–
Recurrent respiratory infections	0	1 (2.2)	0.0038	0	1 (4.5)		0.2367
Allergy	2 (4.3)	1 (2.2)	0.1961	1 (4.5)			0.7839
Atopic dermatitis	3 (6.5)	1 (2.2)	0.3420	0	0		–
Psoriasis	1 (2.2)	0	0.7240	0	0		–
Thyroid disease	1 (2.2)	0	0.7240	0	0		–
Liver diseases	0	0	–	1 (4.5)	0		0.3944
Kidney disease	1 (2.2)	0	0.7240	0	0		–
Cardiologic disease	0	0	–	0	0		–
Diabetes	0	0	–	0	0		–
Hypercholesterolaemia	1 (2.2)	0	0.7240	0	0		–
ADHD	1 (2.2)	0	0.7240	0	0		–
Depression	1 (2.2)	0	0.7240	0	0		–

ADHD, attention deficit hyperactivity disorder.

-, not tested.

## Discussion

In healthy people, Gram-negative bacilli sporadically colonize the oral cavity and are not considered a natural component of the oral microbiota. Their incidence in this site is variable and may increase in some specific conditions, such as immunosuppression, diabetes, oldness, hospitalization, xerostomia, and other oral disorders (Li et al., 2000; Ashreen et al., 2020; Cruz et al., 2022; Hernández-Jiménez et al., 2022). Due to the GNB may cause pneumonia (Levison and Kaye, 1985; Vincent et al., 1995), meningitis (Lanks et al., 2019), bloodstream infection (Cheol-In et al., 2004), endocarditis (Leão et al., 2023), and urinary tract infections (Ceroni, 2013), their presence in the oral cavity poses a potential risk for systemic infections.

In our study, Gram-negative bacilli were frequently isolated from the oral cavity of healthy adolescents, and more than 37% were colonized. Colonization rates worldwide differ considerably depending on the age of the studied population and geographic region. Lower prevalence was reported by Lima et al. in nasopharynx swabs from children attending day-care centers (8.9%) and by Osei et al. in healthy under-five children in Ghana (13.9%) (Lima et al., 2010; Osei et al., 2022). In previous studies from Brazil and Angola, the GNB carriage rates in healthy children were higher and amounted to 50% and 57%, respectively (Wolf et al., 1999). Interestingly, a similar result to ours (32.5%) obtained Gaetti-Jardim Júnior et al. from gingival pockets in a group of HIV-positive adult patients with necrotizing periodontitis (Gaetti-Jardim Júnior et al., 2008).

The most common GNB in our study were *Enterobacter cloacae*, followed by *Pseudomonas* spp. and *Serratia* spp., accounting for 22.1%, 19.1%, and 19.1% respectively. Otherwise, Wolf et al. found a lower prevalence of *Enterobacter cloacae* (5.4%) and *Pseudomonas* spp. (5%) in healthy children (Wolf et al., 2001). The isolation rates of *Enterobacter cloacae* (3.7%) and *Pseudomonas aeruginosa* (0.8%) from nasopharynx in healthy under-five children from day-care centers in Brazil were much lower (Lima et al., 2010). Similar results to ours were reported by authors from Ghana, where *Enterobacter cloacae* was found in 17.5% of healthy children (Osei et al., 2022). Likewise, Aragão et al. isolated *Enterobacter cloacae* from 18.1% of saliva samples of adolescents aged 15 to 19 years from Brazilian public schools (Aragão et al., 2016). Leão et al. highlighted that *Enterobacter cloacae* was the leading pathogen colonizing the oral cavity of healthy workers in intensive care units in a high percentage (46.9%) (Salimiyan Rizi et al., 2019; Leão et al., 2023). The same authors also indicated a similar colonization rate of *Pseudomonas aeruginosa* (18.7%) (Leão et al., 2023).

The origin of Gram-negative bacilli in the oral cavity is not yet clear. Their presence may be due to ingestion of contaminated drinking water and food or poor personal hygiene (Zaatout, 2021). The risk factors may also be socioeconomic status, season, climate, and exposure to environmental pollution (Osei et al., 2022). The analysis of our demographic and clinical data of adolescents showed that oral colonization by GNB was significantly associated with atopic dermatitis and allergic rhinitis. Similarly, Bilal found a relationship between the isolation of GNB from the affected areas and the severity of the course of atopic dermatitis (Bilal et al., 2013). Recently, Paramita showed Gram-negative bacilli as one of the most frequently isolated bacteria from lesions in patients with atopic dermatitis (Paramita et al., 2022).

As stated, GNB being oral colonizers may spread to the respiratory system and trigger life-threatening infections, especially in the hospital setting (Scannapieco et al., 2009). *Pseudomonas aeruginosa* was associated with nosocomial infections that most often affect the lower respiratory system (Kollef et al., 2021). Scannapieco established that a passage of *Pseudomonas* bacteria into the lungs may occur by passive aspiration of the bacterial microbiota released in saliva or eased by medical devices such as bronchoscopes and endotracheal tubes (Scannapieco et al., 2009; Ak et al., 2011). Moreover, *Pseudomonas aeruginosa* is listed as a nosocomial alarm pathogen from the ESKAPE group which includes six multidrug resistant bacteria (Mulani et al., 2019; Venkateswaran et al., 2023). As our study proved, the majority of Gram-negative bacilli, including *Pseudomonas* strains showed multi-drug resistance, above 70%. Alarmingly, the proportion of MDR strains isolated from patients with nosocomial infections reached similar high values (Alkofide et al., 2020; Mirzaei et al., 2021). The commonly used first-line antibiotics in the treatment of *Pseudomonas* infections are carboxypenicillins, ureidopenicillins and their combinations with β-lactamase inhibitors, 3rd and 4th generation cephalosporins, carbapenems, fluoroquinolones, and colistin. In our study, we noted resistance of *Pseudomonas* strains to ticarcillin, piperacillin with tazobactam, ceftazidime, cefepime, imipenem, ciprofloxacin, and colistin, which definitely limits the potential therapeutic options. Studies showed that prolonged antibiotic therapy can lead to the emergence of antibiotic resistance (Yusuf et al., 2017). To our knowledge, multidrug resistant oral strains were isolated without previous antibiotic treatment of adolescents.

In the present study, *Enterobacter cloacae* strains were the predominant GNB-producing ESBLs. The reasons for the increasing prevalence of ESBL strains in many sites are not fully known. The occurrence of ESBL-positive bacilli depends on the geographical area and the regional use of antibiotics. They may be present in contaminated drinking water and improperly discharged sewage (Hawkey, 2008; Dhillon and Clark, 2012). The problem may also be related to the increasing use of antibiotics in veterinary medicine leading to the transmission of ESBL strains from animals to humans (Carattoli, 2008). In many regions of the world, community-acquired ESBL (CA-ESBL) infections are becoming more common and colonization with ESBL-positive strains seems to be underestimated (Tal Jasper et al., 2015). It stated that infections with ESBL were strongly associated with previous colonization (Pena et al., 2001; Troche et al., 2005; Reddy et al., 2007; Bert et al., 2012). Regardless of the site of infection, the main reservoir of ESBL-positive bacteria was the gastrointestinal tract (Donskey, 2004; Bert et al., 2012; Carlet, 2012). Karanika assessed the worldwide average intestinal colonization rate among healthy people at 14% (Karanika et al., 2016). The authors showed that in the Americas the incidence reaches no more than 2%, depending on the region of Europe from 3% to 6%, while in the Asian and African populations, it ranges to 46% (Karanika et al., 2016). Interestingly, our results from the oral cavity seem to be similar (7.4%). Mirzaei et al. found that Gram-negative bacilli isolated from the oral cavity have the ability to colonize the intestines in dysbiosis (Mirzaei et al., 2021). Perhaps it should be considered that the oral cavity is an underestimated reservoir of ESBL-producing bacilli. In our study, the prevalence of ESBL-producing strains was significantly associated with participants reporting recurrent respiratory infections, nasal congestion, and bronchitis. Many authors point to ESBL-GNB colonization as a risk factor for lower respiratory tract infections (van Prehn et al., 2018; Le et al., 2020). Undoubtedly, further studies are needed to fully understand the relationships between oral colonization with ESBL-positive GNB and respiratory infections in adolescents.

The potential limitation of the present study was the lack of follow-up studies in adolescents to verify whether the presence of Gram-negative bacilli in the oral cavity was transient or permanent colonization. Longitudinal studies would be needed to track the presence of these bacteria over an extended period. In addition, all the adolescents in our study were from the same city of Sopot, the study contains some geographical bias. The next one, the study was limited to one age category of participants.

## Conclusions

Despite these limitations, to our knowledge, this is the first study in Poland that revealed that above one-fourth of healthy adolescents carried in their oral cavity multi-drug resistant Gram-negative bacilli. Moreover, oral carriage of ESBL-producing GNB strains was associated with respiratory infections. These findings justify further monitoring of oral colonization by antimicrobial-resistant GNB and identifying the factors responsible for their spread. Knowledge about MDR-GNB oral colonizers may be useful for predicting empirical antibiotic therapy at the risk of potential infection in adolescents.

## Data availability statement

The original contributions presented in the study are included in the article/supplementary material. Further inquiries can be directed to the corresponding author.

## Ethics statement

This study was approved by the bioethics committee of the Medical University of Gdansk (NKBBN/510-745/2021). The studies were conducted in accordance with the local legislation and institutional requirements. Written informed consent for participation in this study was provided by the participants’ legal guardians/next of kin.

## Author contributions

KG: Conceptualization, Funding acquisition, Writing – original draft, Writing – review & editing. MK: Conceptualization, Data curation, Investigation, Methodology, Visualization, Writing – original draft, Writing – review & editing. EK: Conceptualization, Data curation, Investigation, Methodology, Writing – review & editing. KS: Data curation, Funding acquisition, Investigation, Project administration, Writing – review & editing. AK: Supervision, Writing – review & editing. DC: Data curation, Writing – review & editing. DŚ: Software, Writing – review & editing.
